# *Pseudomonas aeruginosa* prosthetic joint-infection outcomes: Prospective, observational study on 43 patients

**DOI:** 10.3389/fmed.2022.1039596

**Published:** 2022-12-08

**Authors:** Héloïse Prié, Vanina Meyssonnier, Younes Kerroumi, Beate Heym, Olivier Lidove, Simon Marmor, Valérie Zeller

**Affiliations:** ^1^Centre de Référence des Infections Ostéo-Articulaires Complexes, Groupe Hospitalier Diaconesses–Croix Saint-Simon, Paris, France; ^2^Service de Médecine Interne et Infectiologie, Groupe Hospitalier Diaconesses–Croix Saint-Simon, Paris, France; ^3^Laboratoire des Centres de Santé et Hôpitaux d’Île-de-France, Groupe Hospitalier Diaconesses–Croix Saint-Simon, Paris, France; ^4^Service de Chirurgie Osseuse et Traumatologique, Groupe Hospitalier Diaconesses–Croix Saint-Simon, Paris, France

**Keywords:** prosthetic joint infection, one-stage exchange, debridement and implant retention, ciprofloxacin, *Pseudomonas aeruginosa*, ceftazidime

## Abstract

**Objectives:**

Analysis the outcomes of *Pseudomonas aeruginosa* prosthetic joint infection (PJI), and of their clinical and microbiological characteristics, surgical strategies and antibiotic treatments.

**Methods:**

Monocenter cohort study in a Bone-and-Joint-Infection Referral Center (08/2004 to 10/2018) including all consecutive *P. aeruginosa* PJIs. Data were extracted from the prospective database, including the following events: relapses, new PJIs, related deaths.

**Results:**

Median [IQR]: among the 43 patients included (28 females; 72 [63–80] years old; 27 hip, 15 knee, and 1 shoulder PJIs), 29 (67%) had underlying comorbidities, 12 (28%) had previously been treated for another PJI and 9 (21%) had undergone previous surgeries for their *P. aeruginosa* PJI. Eleven (26%) PJIs were polymicrobial, 16 (37%) strains were wild type, 8 (19%) ciprofloxacin-resistant. PJIs were classified as late chronic (*n* = 33), early postoperative (*n* = 9) or acute hematogenous infection (*n* = 1). Forty patients underwent surgery: 27 one-stage and 5 two-stage exchanges, 3 debridement and implant retention, and 5 other surgical strategies. Antibiotic treatments were: 29 received 41 [37–43] days of combination therapy (IV anti-pseudomonal β-lactam and 3–5 days of amikacin, then β-lactam and oral ciprofloxacin), followed by oral ciprofloxacin for a total of 12 weeks; 10 received only IV antibiotics for 83 [77–86] days, including 37 [32–46] days of combination therapy; 49 days of ceftazidime alone for 1. During follow-up lasting 33 [24–64.5] months, 2 relapses, 3 new PJIs, and 2 related deaths occurred. Thirty-three (82%) patients and 93% of those managed with one-stage exchange experienced no event.

**Conclusion:**

Outcomes of our cohort’s *P. aeruginosa* PJIs—predominantly monomicrobial, chronic, ciprofloxacin-susceptible, treated with one-stage exchange and prolonged IV antibiotics—were 82% favorable.

## Introduction

Prosthetic joint infection (PJI) is a devastating complication of arthroplasty. The increasing number of joint replacements inevitably leads to increasing PJI incidence, with significant consequences on patients’ morbidity and economic concerns (1–3). Most PJIs are caused by Gram-positive pathogens, while Gram-negative bacilli are found less commonly (11–15%). *Pseudomonas aeruginosa* represents 3–5% of all PJIs (4, 5).

Treatment of *P. aeruginosa* PJIs is considered difficult because of the bacterium’s natural resistance to many antimicrobials, increasing acquired resistance, the nosocomial environment and patients’ comorbidities (6–8). The optimal antibiotic regimen and surgical strategy for *P. aeruginosa* PJIs are not well defined, guidelines are lacking and numerous questions remain.

Only a few retrospective studies focused on *P. aeruginosa* bone-and-joint infections (BJIs) (9–12). One reported 12 cases of early debridement, antibiotics, and implant retention (DAIR)-treated PJIs achieving 75% successes (11). More recently, a French National Referral Center for BJIs reported 90 cases of implant-associated *P. aeruginosa* infections, among which 30 were PJIs (12). Better outcome was associated with intravenous (IV) β-lactam administration for at least 3 weeks and oral ciprofloxacin for 3 months.

This prospective, observational study was undertaken to analyze the outcomes of *P. aeruginosa* PJIs, and to better characterize their clinical and microbiological characteristics, surgical strategies, and antibiotic treatments.

## Methods

### Study design and setting

This monocenter, observational, cohort study was conducted in a French Referral Center for BJIs from August 2004 to October 2018. All patients admitted for a PJI are registered in the Center’s PJI cohort (NCT 019635 and NCT 02801253). Each patient’s epidemiological, clinical, microbiological, therapeutic (surgical and antibiotics), adverse event, and outcome information was entered prospectively. We extracted *P. aeruginosa*-infected patients from the PJI-cohort database. The primary outcome was 2-year event-free survival (EFS).

### Ethics

All patients gave written informed consent, and the cohort was approved by the Île-de-France Ethics Committee.

### Population

All consecutive patients ≥18 years old with confirmed *P. aeruginosa* PJIs were included. *P. aeruginosa* had been isolated from at least two cultures of preoperative joint-fluid and/or intraoperative tissue specimens, and at least one of the following criteria was required: a sinus tract communicating with the prosthesis, local inflammatory signs (swelling, warmth, and erythema), C-reactive protein (CRP) >10 mg/L and/or radiological parameters (i.e., periosteal bone formation and subchondral osteolysis) of infection (3, 4, 13, 14). Polymicrobial infections with *P. aeruginosa* were included.

Prosthetic joint infections were classified on the initial clinical presentation according to three, previously described clinical settings (4, 13) derived from Tsukayama’s classification (15): early-postoperative: onset of symptoms within 30 days after arthroplasty; late-chronic: progressive symptoms occurring ≥30 days after surgery; and acute-hematogenous: defined as sudden onset of local and general symptoms after a symptom-free interval of ≥30 days postsurgery, with identification of a portal of entry or not.

Duration of PJI was calculated from the first day of PJI symptoms to curative surgery in our center, or for non-operated patients to the first day of prolonged suppressive antibiotic surgery.

### Microbiology

*Pseudomonas aeruginosa* and other microorganisms isolated from PJIs were identified in intraoperative sample and/or preoperative joint-fluid aspirate cultures, handled as previously described (4, 14). Bacteria were identified to species with the rapid ID 32 API system (bioMérieux, Marcy-l’Étoile, France) and, since 2012, by mass spectrometry (MALDI Biotyper, Bruker Daltonik GmbH, Bremen, Germany). Antibiotic susceptibilities were determined with the standard disk-diffusion method, according to the recommendations of the French Society of Microbiology (CaSFM) and the European Committee on Antimicrobial Susceptibility Testing (EUCAST).

*Pseudomonas aeruginosa* multidrug resistance (MDR) was defined as previously described (16). When a patient had several different isolates, the most resistant strain was retained for description and choice of treatment.

### Surgery and antibiotic treatments

Medical–surgical management strategies were decided during multidisciplinary consensus meetings, according to the algorithm described in Supplementary Figure 1.

Antibiotics were usually started during surgery after tissue sampling. Preoperative antibiotic therapy was administered systematically until December 2016, and, since January 2017, for selected cases only (clinical sepsis, bacteremia, large local inflammation or abscess, and/or CRP ≥50 mg/L).

Antibiotic therapy lasted 84 days for all patients from 2004 to 2012; 42 days after DAIR; and 84 days after exchange-arthroplasty-treated patients from 2013 to 2016. Since 2017, all patients have received 42 days of antibiotics, except those at high risk of relapse (multiple operations, severe immunosuppression, sickle-cell anemia, irradiated bone, and large bone graft) who were treated for 84 days.

First-line, IV-administered, combination antibiotic therapy (CAT) was ceftazidime–amikacin for 3–5 days, followed by IV ceftazidime and oral ciprofloxacin to complete 28–42 days. Patients treated for 84 days received an additional 42–56 days course of oral ciprofloxacin. In the case of contraindication or resistance to ciprofloxacin, IV CAT with an anti-pseudomonal β-lactam (ceftazidime, cefepime, or meropenem) and fosfomycin, colistin, or aminoglycoside (maximum 2 weeks) was given. High-dose β-lactams (ceftazidime, 100 mg/kg/day, after a 2-g loading dose) were administered by continuous IV infusion to all patients. Drug-monitoring assured reaching target, plasma ceftazidime concentrations of 50–70 mg/L.

Adverse drug-reaction severity was graded according to the Common Terminology Criteria for Adverse Events (CTCAE) (17).

### Outcomes

The primary endpoint was 2-year follow-up event-free-survival (EFS), considering the following events: relapse with the same bacteria, new infection (microorganism different than the initial PJI) and infection- or treatment-related death.

Patients were prospectively followed after discharge at 3, 6, 12, and 24 months, then every 2 years. Patients unable to attend follow-up visits were contacted by phone to assess their health and prosthesis evolution.

### Statistical analyses

Categorical variables are expressed as number (%) and quantitative variables as median [interquartile range (IQR)]. Fisher’s exact test, Student’s *t*-test, or Mann–Whitney U-test, as appropriate, were used to compare PJI baseline admission to our center characteristics and outcomes. The Kaplan–Meier method was used to estimate cumulative EFS incidence and 95% confidence intervals (CIs).

## Results

### Study population and prosthetic joint infection characteristics

During the 14-year study period, among 1,539 managed PJIs, 170 (11%) Gram-negative and 43 (2.8%) *P. aeruginosa* PJIs occurred. Table 1 reports their clinical and laboratory characteristics. Among these 43 patients, 32 (74%) were monomicrobial PJIs. Most were hip and knee PJIs. Patients had undergone a median [IQR] of 2 (1–3) surgeries on the affected joint. More than one-quarter had a previous, other-site PJI. Two-third of the patients had underlying comorbidities detailed in Table 1.

**TABLE 1:** Baseline characteristics of 43 patients with *P. aeruginosa* PJIs and according to type of infection.

Characteristic	All PJIs	Monomicrobial	Polymicrobial	*p*-value
	(*n* = 43)	PJIs (*n* = 32)	PJIs (*n* = 11)	
Female	28 (65)	20 (63)	8 (73)	0.72
Age, years	72 [63–80]	70 [63–77]	75 [70–83]	0.31
PJI site				
Hip	27 (63)	21 (66)	6 (55)	0.72
Knee	15 (35)	10 (31)	5 (46)	0.47
Shoulder	1 (2)	1 (3)	0	1
Underlying comorbidity	29 (67)	19 (59)	10 (91)	0.07
Obesity, body mass index >30 kg/m^2^	15 (35)	10 (31)	5 (46)	0.47
Renal failure^a^	9 (21)	5 (16)	4 (36)	0.2
Diabetes mellitus	7 (16)	4 (13)	3 (27)	0.35
Immunosuppression^b^	4 (9)	3 (9)	1 (9)	1
Heart failure	5 (12)	4 (13)	1 (9)	1
Rheumatoid arthritis	3 (7)	3 (9)	0	0.56
Respiratory failure	1 (2.)	0	1 (9)	0.26
Cirrhosis	1 (2)	0	1 (9)	0.26
Previous PJI	12 (28)	8 (25)	4 (36)	0.47
The same joint	8 (19)	5 (16)	3 (27)	0.4
Another joint	4 (9)	3 (9)	1 (9)	1
Previous extraarticular *P. aeruginosa* infection^c^	7 (16)	5 (16)	2 (18)	1
Previous surgeries for *P. aeruginosa* PJI^d^	9 (21)	6 (18)	3 (27)	0.67
Superficial debridement	3 (7)	2 (6)	1 (9)	1
DAIR	5 (12)	3 (9)	2 (18)	0.59
Two-stage exchange	1 (2)	1 (3)	0	1
PJI classification				
Early postoperative	9 (21)	3 (9)	6 (55)	0.0044
Late chronic	33 (77)	28 (88)	5 (46)	0.0096
Acute hematogenous	1 (2)	1 (3)	0	1
Clinical, at admission				
ASA score				
1 or 2	13 (30)	11 (34)	2 (18)	0.46
3 or 4	30 (70)	21 (66)	9 (82)	0.46
Fever	6 (14)	5 (16)	1 (9)	1
Fistula	15 (35)	11 (34)	4 (36)	1
White blood-cell count^e^	8.1 [7–9]	7.8 [7–9]	8.7 [8–10]	0.17
C-reactive protein^e^	47 [24–86]	36 [23–75]	89 [73–105]	0.014

Results are expressed as *n* (%) or median [interquartile range]. PJI, prosthetic joint infection; DAIR, debridement, antibiotics, and implant retention; ASA, American Society of Anesthesiologists.

^a^Glomerular filtration rate estimated with Modification of Diet in Renal Disease formula <60 ml/min/1.73 m^2^.

^b^Immunosuppressive treatment, active malignancy, uncontrolled human immunodeficiency virus infection.

^c^Urinary tract infections, hematomas, osteosynthesis material infections.

^d^Prior orthopedic surgeries to treat *Pseudomonas aeruginosa* PJI before current treatment.

^e^Laboratory findings at admission to our unit for the current infection.

Only one acute-hematogenous infection was observed. The source of infection was the urinary tract, as the patient experienced urinary sepsis, fever, and bacteremia with the same *P. aeruginosa*, 3 weeks before the onset of PJI signs. Nine other patients developed an early postoperative PJI. Most patients (*n* = 33, 77%) had a late-chronic PJI. The source of infection was considered to be the surgical site, but 7 patients had previous extra articular *P. aeruginosa* infection or colonization. Four involved the urinary tract in the 6 months prior to PJI (pyelonephritis, cystitis, and two urinary tract colonization), two experienced an infected hematoma following arthroplasty and one patient had had an implant-associated infection after osteosynthesis of a femoral fracture infection 25 years ago.

Median [IQR] duration of PJI symptoms before curative surgery or prolonged suppressive antibiotic therapy in our center, was 176 [60–342] days for the total population. It was 53 [20–136] days for early-postoperative and 251 [136–371] days for late-chronic PJIs. For the only hematogenous infection, symptoms lasted 104 days before surgical treatment.

### Microbiological findings

Among 37 joint-fluid cultures of preoperative aspirates, 36 yielded *P. aeruginosa* and one *Staphylococcus simulans*. Cultures of intraoperative samples taken from 40 patients isolated *P. aeruginosa* exclusively in 28 (70%) patients, 11 (28%) were polymicrobial, and 1 from a patient given preoperative antibiotic therapy remained sterile.

Susceptibility testing of the 43 strains showed that 16 (37%) were wild-type *P. aeruginosa*, 35 (81%) were ciprofloxacin-susceptible (Supplementary Table 1), and 4 (9%) were MDR.

The 11 (26%) patients with polymicrobial PJIs were slightly older, had more comorbidities, higher CRP levels and more frequent early-postoperative PJIs (Table 1). Other coinfecting microorganisms were mostly Gram-positive cocci [five each: methicillin-susceptible (MS) coagulase-negative staphylococci (CNS) or *Enterococcus* spp.; four each: MS *Staphylococcus aureus* (MSSA) or Enterobacteriaceae; and two each: methicillin-resistant CNS, *Streptococcus* spp. or Gram-positive bacilli].

### Surgical strategies

Three patients with early-postoperative PJIs underwent DAIR 13, 28 or 30 days after prosthesis implantation.

Thirty-two patients underwent prosthesis exchange. Twenty-seven were one-stage exchanges: 5 were polymicrobial PJIs, 24 ciprofloxacin-susceptible, and 2 MDR *P. aeruginosa* strains.

Other surgical treatments were knee arthrodeses and hip resections (two each), in patients with previous, same-site PJIs. One patient underwent transfemoral amputation for knee PJI with sepsis and extensive skin necrosis.

Polymicrobial infections were more frequently managed with DAIR or other surgery (6/11, 55%), rather than with prosthesis exchange (5/11, 46%), compared to monomicrobial PJIs (7 and 93%, respectively, *p* = 0.003).

The four MDR-*P. aeruginosa* PJI patients underwent one-stage for 2, and two-stage exchange or hip-resection, for one each.

Three non-operated patients were treated with prolonged suppressive antibiotic therapy (PSAT).

### Antibiotic strategy

#### Preoperative antibiotics

Among 30 (75%) patients given preoperative IV antibiotics, 24 (80%) received combined β-lactam and aminoglycoside (mostly ceftazidime and amikacin, for 21) for a median [IQR] of 4.5 (2–6) days. The others received β-lactam monotherapy or association with fosfomycin or colistin.

#### Postoperative curative antibiotics

Types, molecules and durations of antibiotic treatments are reported in Table 2. Among 29 (73%) patients receiving first-line antibiotics, 24 (83%) underwent prosthesis exchange, 22 of which were one-stage. Eleven patients were prescribed alternative antibiotic regimens: seven with ciprofloxacin-resistant strains and four with ciprofloxacin contraindications. Thirty-one (78%) patients received antibiotics for 84 days and nine (23%, including the three DAIR-treated) for 42 days.

**TABLE 2:** Post-operative antibiotic therapies and outcomes of 40 *P. aeruginosa* PJIs after curative surgery.

Treatment	Value
Surgical strategy	
DAIR	3 (8)
Exchange arthroplasty	32 (80)
One-stage exchange	27 (68)
Two-stage exchange	5 (13)
Other strategies	5 (13)
Antibiotic therapy	
First-line therapy^a^, including	29 (73)
Combination therapy, including	29 (73)
Ceftazidime	24 (83)
Cefepime	1 (3)
Piperacillin–tazobactam	4 (14)
Aminoglycoside	14 (48)
Ciprofloxacin	29 (100)
Additional oral ciprofloxacin monotherapy	29 (73)
Days of combination therapy	41 [37–43]
Total days of treatment	84 [82–84]
Only IV combination antibiotic therapy^b^, including	10 (25)
Anti-pseudomonal β-lactam	10 (100)
Aminoglycoside	4 (40)
Fosfomycin	8 (80)
Colistin	2 (20)
Days of combination therapy	36 [31–45]
Total days of treatment	83 [77–86]
Ceftazidime monotherapy	1 (3)
Total days of treatment	49
Serious adverse antibiotic-related event^c^	3 (8)
Outcome	
Duration of follow-up, in months	33 [24–64.5]
Events	7 (18)
Related death	2 (5)
Relapse	2 (5)
New infection	3 (8)

Results are expressed as *n* (%) or median [interquartile range]. PJI, prosthetic joint infection; DAIR, debridement, antibiotics, and implant retention.

^a^As described in section “Surgery and antibiotic treatment.”

^b^Exclusively IV antibiotic therapy, including time on bitherapy: anti-pseudomonal β-lactam with amikacin + another anti-pseudomonal class of antibiotic.

^c^Grade ≥3 according to Common Terminology Criteria for Adverse Events.

Three patients experienced antibiotic serious adverse events (grade ≥3): acute renal failure, esophageal candidiasis, and *Clostridium difficile* colitis. None required antibiotic discontinuation.

#### Prolonged suppressive antibiotic therapy

Three very old and frail patients received PSAT comprising oral ciprofloxacin after 19–28 days of IV ceftazidime with amikacin or without. Drug-induced peripheral neuropathy forced two to stop ciprofloxacin after 6 and 18 months.

### Outcomes

Median [IQR] follow-up lasted 33 [24–64.5] months for the 40 patients managed with a curative strategy; two were lost-to-follow-up before 24 months.

**FIGURE 1:**
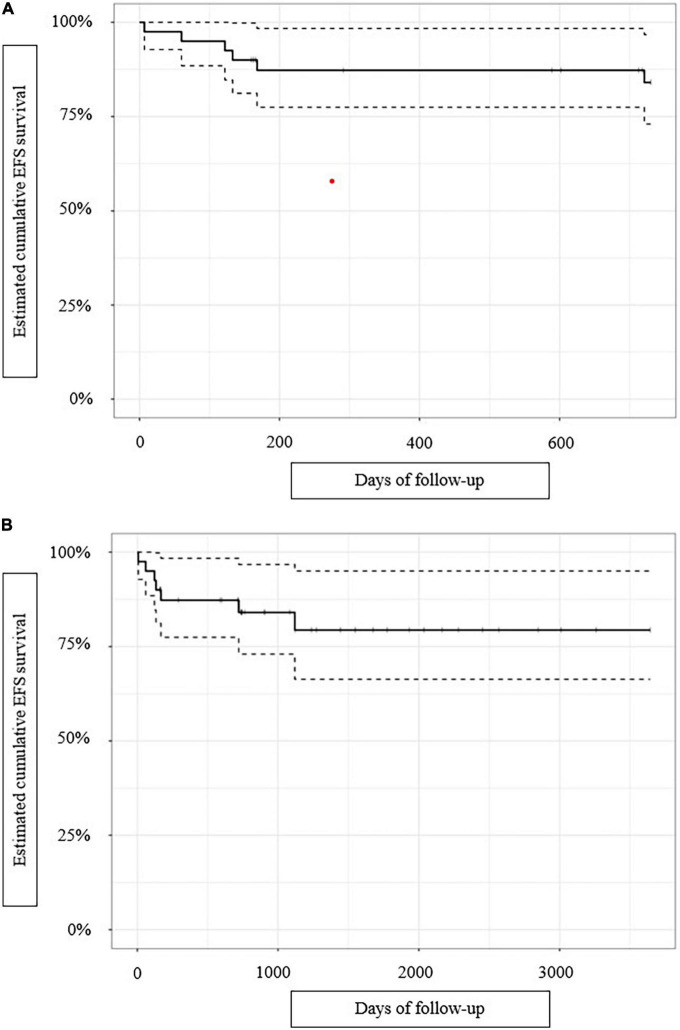
For 40 patients curatively treated for *P. aeruginosa* prosthetic joint infections, Kaplan–Meier-estimated: **(A)** cumulative 2-year event-free survival (EFS), and **(B)** cumulative end-of-follow-up event-free survival. The dashed lines indicated the confidence intervals.

Seven events occurred: two related deaths, three new PJIs, and two relapses (Table 3). The 2-year and end-of-follow-up crude EFS rates were 85 and 82%, respectively. The estimated 2-year and end-of-follow-up cumulative EFS rates (CIs) were 84% (73–96.7%) and 79.4% (66.3–95%), respectively (Figure 1). For patients managed with one-stage exchange arthroplasty, the 2-year and end-of-follow-up crude EFS rates were 96 and 92%, respectively.

**TABLE 3:** Details of the reported events.

					Antibiotic therapy						
	Age			Initial	Bitherapy		Monotherapy		Total		Surgery-to-event
No.	Year	Infection type	PJI type	Surgery	Molecule	Days	Molecule	Days	days	Event: details	Days
1	86	Monomicrobial	Chronic	1SE	MEM + CIP	47	CIP	44	91	Related death: failure to thrive	123
2	77	Polymicrobial	Early postoperative	Amputation	MEM + AN then CIP	40	CIP	20	60	Related death: septic shock	60
3	83	Polymicrobial	Early postoperative	DAIR	TZP + AN then CIP	41	–	0	41	Relapse: *Pseudomonas aeruginosa*	710
4	70	Monomicrobial	Chronic	1SE	CAZ + CIP	40	CIP	43	83	Relapse: *Pseudomonas aeruginosa*	1118
5	67	Polymicrobial	Chronic	Resection	CAZ + CL	33	CAZ	57	90	New PJI: *Enterococcus faecium, Enterococcus gallinarum*	7
6	53	Polymicrobial	Chronic	Resection	CAZ + CIP	23	CIP	61	84	New PJI: *Finegoldia magna, MSSA*	133
7	66	Monomicrobial	Chronic	2SE	CAZ + CIP	62	CAZ	22	84	New PJI: *E. faecalis, MSSA*	168

1SE, one-stage exchange; 2SE, two-stage exchange; DAIR, debridement and implant retention; MEM, meropenem; CIP, ciprofloxacin; AN, amikacin; TZP, piperacillin–tazobactam; CAZ, ceftazidime; CL, colimycin; MSSA, methicillin-sensitive *Staphylococcus aureus*.

Among the three DAIR-treated patients, an 83-year-old diabetic man, with chronic renal insufficiency, and wild-type *P. aeruginosa* and MSSA hip PJI operated 28 days after prosthesis implantation, relapsed with a persistent wild-type strain.

Among 32 patients who underwent prosthesis exchange, three (9%) experienced events: a 70-year-old woman with no comorbidity after chronic *P. aeruginosa* knee PJI relapsed with the same wild-type *P. aeruginosa*; one related death (multiple prolonged decubitus complications within 1 year) and a new PJI (MSSA and *Enterococcus faecalis*).

One 77-year-old patient died of septic shock after urgent knee amputation for necrotizing polymicrobial PJI. Two patients who underwent hip resection for chronic polymicrobial PJIs developed new polymicrobial PJIs.

Event-free survival tended to be lower for patients with polymicrobial than monomicrobial PJIs (64 vs. 90%, *p* = 0.075). It was comparable for patients with ciprofloxacin-susceptible (84%; five events: two relapses, one new PJI, and two related deaths) and ciprofloxacin-resistant PJIs (75%; two new PJIs).

Two of the three PSAT-treated patients relapsed 7 months and 2 years after having stopped ciprofloxacin for adverse events; their relapsing strains remained ciprofloxacin-susceptible.

## Discussion

We described 43 *P. aeruginosa*-PJI patients extracted from a prospective cohort and managed in a BJI Referral Center over 14 years. The cohort’s strength is its focus on PJIs and its prospective, prolonged follow-up.

These patients included a high percentage with previous PJIs and surgeries, as also observed by others, partly explained by the nosocomial origin of *P. aeruginosa* (9, 10, 18). Unlike previous studies, three-quarters of our observed PJIs were late-chronic, monomicrobial infections, whereas Gram-negative and *P. aeruginosa* PJIs are often early-postoperative and polymicrobial (5, 11, 12, 19). Our Referral Center’s BJI activity has certainly contributed to the selection of this different PJI population, because 21% of our patients had been transferred from primary-care centers after undergoing a first surgical intervention that failed. These late chronic PJIs seem to be acquired per or postoperatively, but among them 7 had a history of previous extra articular, especially urinary, *P. aeruginosa* infection or colonization. This raises the question of whether these extra articular infections are not the PJI source, finally acquired by hematogenous route or by contiguity with the infectious site.

Overall, 85 and 82% of the operated patients had not experienced any event at 2-years and the end-of-follow-up, respectively; rates higher than the 68–75% previously reported (9, 12, 20). Our patients’ outcomes differed according to the surgical strategy. For prosthesis exchange, the most frequent strategy applied in our cohort, EFS reached 91% and even 93% when considering only one-stage exchanges. Only one patient relapsed. These results are highly satisfying and encouraging, especially when compared to the largest *P. aeruginosa*-PJI population described by Shah et al. (9). Those authors had a 5-year 71% failure-free rate after one-stage and 39% after two-stage exchange. Among our three DAIR-treated patients with early-postoperative PJIs, one relapsed. No definitive conclusions can be drawn based on such small numbers, but post-DAIR failure-free survival reported by others ranged between 26 and 79%, close to our values (9, 11, 20).

We prescribed prolonged antibiotic therapy with 42-days-long CAT with IV β-lactam and oral ciprofloxacin for nearly three-quarters of our patients. Ceftazidime was the first-choice β-lactam, because of its excellent bactericidal activity against *P. aeruginosa* (21, 22). Data on ceftazidime bone diffusion are scarce, reportedly 20–40% of the serum concentration (23). To optimize ceftazidime’s time-dependent activity and reach therapeutic concentration, we used continuous, high-dose infusion adapted by drug-monitoring, as previously supported about antipseudomonal β-lactam in general (24).

Amikacin was combined for the first few days to avoid development of drug resistance and enhance bactericidal activity, followed by oral ciprofloxacin, also controlled by drug-monitoring. Ciprofloxacin alone was prescribed for 42 additional days, to patients treated for 84 days.

In their recent study, Cerioli et al. (12) observed that effective IV β-lactam lasting at least 3 weeks and ciprofloxacin for at least 3 months were independently associated with better outcomes. Other authors also observed better outcomes when fluoroquinolones were prescribed to treat *P. aeruginosa* and Gram-negative PJIs (12, 20, 25), attributed to their high bone diffusion and activity in biofilm (26, 27). Optimal durations of initial IV and CAT remain unknown, but Cerioli et al.’s findings and our results support prolonged, combined, IV β-lactam and ciprofloxacin, longer than that usually accepted for fluoroquinolone-susceptible Enterobacteriaceae PJIs (20, 25).

The 20% ciprofloxacin-resistance rate observed in our population was not high and close to that reported for invasive *P. aeruginosa* strains in France (28). No standard treatment is recommended for ciprofloxacin-resistant *P. aeruginosa* PJIs. The eight patients with ciprofloxacin-resistant strains received 12 weeks of IV antibiotics, including 5 weeks of CAT; none relapsed but two developed new PJIs.

The three PSAT-treated patients had favorable clinical and microbiological outcomes until they stopped ciprofloxacin because of adverse events. Fluoroquinolone-susceptible strains were also found at relapse. This suppressive therapeutic option should be chosen only when curative strategies are contraindicated. Indeed, ciprofloxacin is the only fluoroquinolone available, and its use is limited by potential resistance emergence and adverse events under prolonged monotherapy. More data are needed to determine whether PSAT with ciprofloxacin is indeed a therapeutic option (29, 30).

Outcomes of polymicrobial PJIs were poorer than monomicrobial infections. Although not previously identified as a risk factor for treatment failure, polymicrobial infections were mostly treated with DAIR or non-conservative surgery, strategies that have poorer outcomes than prosthesis exchange.

This study had several limitations, especially the small size of the population. However, *P. aeruginosa* PJIs are quite rare and the single-center design precluded multivariate analyses. Because of Referral Center selection bias, results are difficult to extrapolate to other populations, particularly those with higher MDR rates. We did not collect the ambulatory status of the patients during the follow-up, which prevent us from evaluating the functional results of medico-surgical treatment. Notably, the study period was long and procedures evolved over time, leading to different PJI treatments. However, observational studies can yield precise data on rare and specific situations, helping to optimize management of this devastating nosocomial complication.

## Conclusion

In addition to global favorable outcomes for 82% of these difficult-to-treat PJIs, we observed >90% favorable outcomes for the main population managed with one-stage exchange and prolonged IV antibiotics for chronic ciprofloxacin-susceptible *P. aeruginosa* PJIs. Additional larger studies are needed to better specify optimal surgical strategies and antibiotics.

## Data availability statement

The raw data are not publicly available because French regulatory and Ethics Committees do not authorize the deposition of personal patient data in a public database. Requests to access the datasets should be directed to the corresponding author.

## Ethics statement

The studies involving human participants were reviewed and approved by the Île-de-France Ethics Committee. The patients/participants provided their written informed consent to participate in this study.

## Author contributions

HP, VM, VZ, YK, and SM conceived the study. HP, VM, YK, and VZ analyzed the data. HP, VM, and VZ wrote the original draft of the manuscript and performed the literature review. VM and VZ reviewed and edited the manuscript. VZ, SM, and YK were the investigators of the prospective cohort. VM, VZ, BH, OL, and SM participated in the patient care and the review of the manuscript. All authors contributed to the article and approved the submitted version.
