# Supplementary material for: Pseudomonas aeruginosa prosthetic joint-infection outcomes: Prospective, observational study on 43 patients
Source: Front Med (Lausanne). 2022 Dec 8;9:1039596. doi: 10.3389/fmed.2022.1039596 (PMC9774483; doi:10.3389/fmed.2022.1039596)
Supplement: Supplementary file 1 [file Data_Sheet_1.PDF]

Figure 1 (supplementary material). Surgical strategies

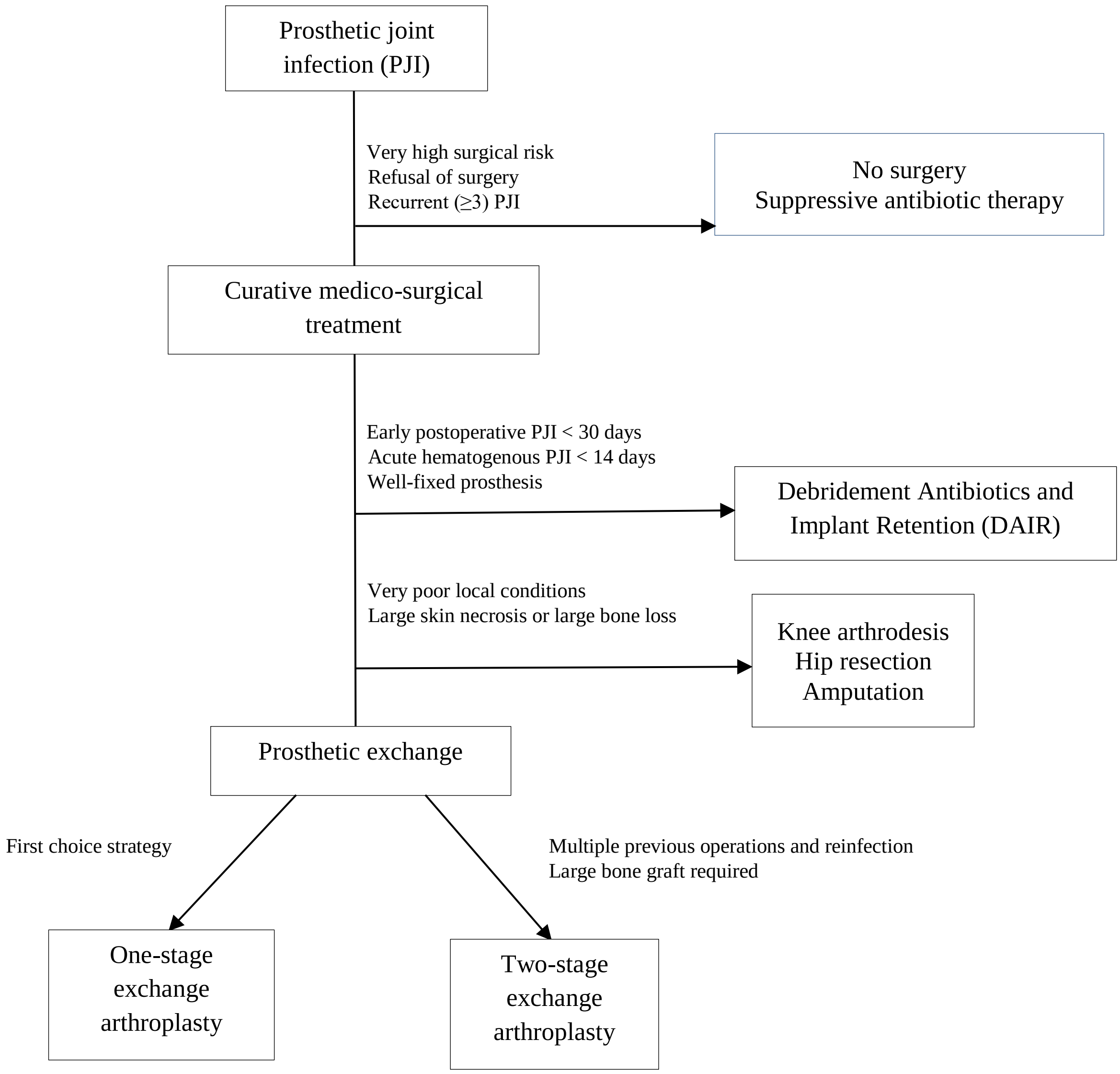

Table 1. In vitro susceptibilities of selected antimicrobials against *Pseudomonas aeruginosa* isolates in 43 strains

Antimicrobial Agent	Susceptibility	
	n	%
Piperacillin-tazobactam	38	88,4
Cefepime	39	90,7
Ceftazidime	41	95,3
Meropenem	38	88,4
Imipenem	36	83,7
Ciprofloxacin	35	81,4
Gentamicin	36	83,7
Amikacin	41	95,3
Tobramycin	38	88,4
Fosfomycin	29	67,4
Colistine	43	100
